# Stem–Mesenchymal Signature Cell Genes Detected in Heterogeneous Circulating Melanoma Cells Correlate With Disease Stage in Melanoma Patients

**DOI:** 10.3389/fmolb.2020.00092

**Published:** 2020-05-29

**Authors:** Maria Cristina Rapanotti, Elena Campione, Tara Mayte Suarez Viguria, Giulia Spallone, Gaetana Costanza, Piero Rossi, Augusto Orlandi, Piera Valenti, Sergio Bernardini, Luca Bianchi

**Affiliations:** ^1^Department of Onco-Haematology, Tor Vergata University of Rome, Rome, Italy; ^2^Department of Experimental Medicine, Tor Vergata University of Rome, Rome, Italy; ^3^Department of Dermatology, Tor Vergata University of Rome, Rome, Italy; ^4^Anatomic Pathology Division, Department of Biomedicine and Prevention, Tor Vergata University of Rome, Rome, Italy; ^5^Surgery Division, Department of Systems Medicine, Tor Vergata University of Rome, Rome, Italy; ^6^Department of Public Health and Infectious Diseases, Sapienza University of Rome, Rome, Italy

**Keywords:** liquid biopsy, circulating melanoma cells, MCAM/MUC18/CD146, ABCB5, gene-expression panel, melanoma disease progression

## Abstract

During the process of metastasis, cancer cells dissociate from primary tumors, migrate to distal sites, and finally colonize, eventually leading to the formation of metastatic tumors. These cancer cells, defined circulating tumor cells (CTCs) spreading through the blood stream, may develop metastatic lesions or remain dormant. Some emerging clinical evidence supports that some tumor cells may possess metastatic properties already in the earlier stages of tumorigenesis. Because the initiation and progression of vertical growth in human melanoma is fundamental to the notion of tumor virulence and progression, we decided to immune-magnetic collect and molecularly characterize circulating melanoma cells (CMCs) from melanoma patients AJCC staged = pT1b (i.e., transition from radial to vertical phase). CMCs are phenotypically and molecularly heterogeneous, thus we performed a “home-made Liquid-Biopsy,” by targeting the melanoma-associated-antigen, MCAM/MUC18/CD146, and/or the melanoma-initiating marker, ABCB5. We assessed a biomarker qualitative expression panel, contemplating the angiogenic-potential, melanoma-initiating and melanoma-differentiation drivers, cell-cell adhesion molecules, matrix-metallo-proteinases, which was performed on three enriched subpopulations from a total of 61 blood-samples from 21 melanoma patients. At first, a significant differential expression of the specific transcripts was documented between and within the CMC fractions enriched with MCAM-, ABCB5-, and both MCAM/ABCB5-coated beads, when analyzing two distinct groups: early AJCC- (stage I–II) and advanced- staged patients (stage II–IV). Moreover, in the early-AJCC staged-group, we could distinguish “endothelial,” CD45^–^MCAM^+^ enriched-, “stem” S-CMCs, CD45^–^ABCB5^+^ enriched- and a third hybrid bi-phenotypic CD45^–^MCAM^+^/ABCB5^+^ enriched-fractions, due to three distinct gene-expression profiles. In particular, the endothelial-CMCs were characterized by positive expression of genes involved in migration and invasion, whilst the stem CMC-fraction only expressed stem and differentiation markers. The third subpopulation isolated based on concurrent MCAM and ABCB5 protein expression showed an invasive phenotype. All three distinct CMCs sub-populations, exhibited a primitive, “stem-mesenchymal” profile suggesting a highly aggressive and metastasizing phenotype. This study confirms the phenotypic and molecular heterogeneity observed in melanoma and highlights those putative genes involved in early melanoma spreading and disease progression.

## Introduction

At present, we still know little about tumor progression once metastasis is initiated. Therefore, more should be learned about metastasis initiation at cellular and molecular level, as this could provide predictions on timing and targets of antimetastatic therapies (Coghlin and Murray, 2010). Metastasis is traditionally considered a relatively late process in tumor progression as this process is often diagnosed in primary tumors at a later stage (Luzzi et al., 1998; Páez et al., 2012). During the metastasis process, cancer cells dissociate from primary tumors, migrate to distal sites, and finally colonize, and eventually form metastatic tumors. These cancer cells—known as circulating tumor cells (CTCs)—spread through the blood stream and may develop metastatic lesions or remain dormant (Paterlini-Brechot and Benali, 2007; Sosa et al., 2014). The concept of dormancy corresponds to the existence of an early or late pause (lag time) period in progression; that is, it may occur at an early as well as at a late stage of tumor development and relapse, either locally or at distant sites (Haass et al., 2005; Mocellin et al., 2006). Functionally, when dormant, disseminated tumor cells persist particularly in a stable, non-dividing state of quiescence rather than in a balanced proliferation (Haass et al., 2005; Mocellin et al., 2006). Even small cancers (<5 mm in diameter) may potentially lead to multiple metastases 5–10 years before they are detected. The early-disseminated cells might not turn into overt metastases but remain quiescent for a long time (Friberg and Nyström, 2015; Hanahan and Weinberg, 2011; Rocken, 2010).

It is generally thought that metastasis-initiating cells originate from a subpopulation present in progressed, invasive tumors (Györgyi and Ferenc, 2017); however, there is emerging clinical evidence supporting that some tumor cells may possess metastatic properties in the earlier stages of tumorigenesis (Husemann et al., 2008). The recent application of genomic approaches in determining molecular signatures also suggests that metastasis capacity may be acquired at the initial stages of tumor development (Bernards and Weinberg, 2002). Disseminated cancer cells detected in patients before the manifestation of breast-cancer metastasis contain fewer genetic abnormalities than primary tumors or than tumors of patients with metastases (Klein et al., 1999; Schmidt-Kittler et al., 2003; Schardt et al., 2005). These findings, and others documented in pancreatic cancer (Rhim et al., 2012) and melanoma (Eyles et al., 2010) models, indicate that dissemination might occur during the early stages of tumor evolution (Schardt et al., 2005; Husemann et al., 2008; Sänger et al., 2011; Harper et al., 2018). However, the mechanisms that might allow early-disseminated cancer cells to complete all steps of metastasis are unknown.

Liquid biopsy is renowned as a non-invasive test to detect CTCs or the products of tumors, including proteins, cell-free DNA, and exosomes, and is an interesting tool to facilitate cancer research and patient monitoring. Circulating tumor cells, with respect to other materials, have some advantages in clinical applications; they can be identified morphologically, and their genetic and molecular characterization can be performed by analyzing both specific selected DNA mutations and potential tumor biomarkers (Pantel and Alix-Panabières, 2013, 2016). In particular, CTC biological signatures, such as epithelial-to-mesenchymal transition (EMT) or cancer stemness, may also be analyzed. EMT, which is indispensable for tumor metastasis, is a multistep process involving many molecular and cellular changes, including the downregulation of epithelial proteins and the upregulation of mesenchymal proteins, endowing the cells with increased motility and invasiveness (Kalluri and Weinberg, 2009; Thiery et al., 2009). Recent studies have revealed that the EMT phenotype in CTCs may facilitate tumor metastasis. Characterizing the epithelial versus mesenchymal phenotypes of CTCs may be useful to identify the most aggressive CTC subpopulations and establish an appropriate therapy (Zehentner et al., 2006; Lembessis et al., 2007; Tsouma et al., 2008; Theodoropoulos et al., 2010; Yu et al., 2013; Okabe et al., 2014; Satelli et al., 2015; Zhao et al., 2019). Because of the aggressiveness and mortality of metastatic melanoma cancer, it has become increasingly urgent to define novel diagnostic melanoma biomarkers that can be useful to predict an increased risk of metastasis at an early stage. To date, the lactic dehydrogenase enzyme is the only peripheral blood biomarker incorporated in the American Joint Committee on Cancer (AJCC) classification (Gershenwald and Scolyer, 2018), considering that elevated serum lactate dehydrogenase levels are associated with an unfavorable translation prognosis.

In malignant melanoma (MM), CTCs are detectable in the peripheral blood soon after the surgical resection of the primary tumor regardless of the thickness, or even in late stages or in clinically disease-free patients (Rose et al., 1986; Tsukamoto et al., 1992; Gaugler et al., 1994; Ghossein and Rosai, 1996; Mellado et al., 1999; Palmieri et al., 1999; Keilholz et al., 2004). Measuring circulating melanoma cells (CMCs) before they become clinically detectable represents a potentially powerful method to monitor patients with malignancies that have a minimal morbidity. For melanoma, two studies showed that the number of “2 CMCs per 7.5 mL of blood” is prognostic and associated with shorter survival (Rao et al., 2011; Klinac et al., 2014). In carcinomas, immunomagnetic enrichment is conventionally performed targeting the epithelial cells with surface markers such as EpCAM and cytokeratin antigens as “positive” selection. The Food and Drug Administration has approved the CellSearch^®^ Circulating Tumor platform for the collection and isolation of CTCs of these carcinomas. At present, only EpCAM (Janssen Diagnostic, LLC Raritan, NJ, United States) has been recognized. As already reported, CMCs lack a ubiquitous marker because they do not express the classic epithelial cell surface marker EpCAM due to the origin of the melanocytes from the neural crest. Nevertheless, a variety of markers associated with some melanoma-specific cell-surface epitopes have been proposed, such as MCAM/MUC18/CD146 and MSCP/NG2 (melanoma-associated chondroitin sulfate), together with stem cell markers, such as ABCB5 (ATP-binging cassette subfamily member B) and CD271 (Boiko et al., 2010; Khoja et al., 2013; Luo et al., 2014).

The cell–cell adhesion molecule MCAM/MUC18/CD146, expressed up to 80% in MM, is a key oncogene driving melanoma progression and metastasis (Ishikawa et al., 2014a, b). We previously documented that MCAM/MUC18/CD146 expression predicts clinical relapse, whereas absence or persistent loss is linked to stable disease or disease-free status, revealing its possible role as “molecular warning of progression” (Rapanotti et al., 2009, 2013, 2014, 2017; De Luca et al., 2017). Recently, two MCAM/MUC18/CD146 isoforms have been described, a long and a short variant due to alternative splicing: the short isoform is widely expressed by the endothelium, whereas the long isoform is expressed by melanoma cells. In addition to the membrane-anchored MCAM/MUC18/CD146, a soluble form—sCD146/MCAM/MUC18—generated by metalloproteases proteolytic cleavage is mainly involved in tumor angiogenesis. Expressing MCAM/MUC18/CD146-positive tumors secrete soluble CD146 that, in turn, are responsible for their growth and vascularization (Alais et al., 2001; Stalin et al., 2013, 2017).

Another functional driver of melanoma aggressiveness features through a common molecular role in tumor growth, maintenance, and drug resistance is exerted by the cell membrane-associated transporter ABCB5 (ATP-binding cassette sub-family B member 5, also known as P-glycoprotein) (Frank et al., 2005; Schatton et al., 2008). Human tumorigenic melanoma reveals that few melanoma cells express ABCB5. These cells tend to display a primitive molecular profile and correlate with clinical melanoma progression as determined by high-density tissue microarrays that allow one to screen many melanomas representing progressive evolution from radial [radial growth phase (RGP)] to growth phase [vertical growth phase (VGP)] (Klein et al., 2007) and metastatic disease. Thus, this plasma membrane-spanning protein that behaves as “stem cell” marker of a slow-cycling population of malignant cell subpopulations with “clinical virulence resides” as a consequence of unlimited self-renewal, resulting in inexorable tumor progression and metastasis (Boiko et al., 2010; Laga et al., 2010; Civenni et al., 2011; Kupas et al., 2011; Linley et al., 2012; Tang et al., 2012), could represent a melanoma biomarker of early metastatic stage.

All these findings strongly support us to improve CMC detection in order to investigate MCAM/MUC18/CD146 and ABCB5 as enrichment markers in early and widespread metastasis. Because the initiation and progression of vertical growth in human melanoma are fundamental for the notion of tumor virulence and progression, we decided to collect and analyze CMCs from melanoma patients AJCC stage > pT1b (i.e., transition from radial to vertical phase), improving highly aggressive cell recovery of three subpopulations based on MCAM/MUC18/CD146, ABCB5, or biphenotypic MCAM/MUC18/CD146-ABCB5 expressions.

Consequently, we assessed a selected biomarker qualitative expression panel, contemplating the angiogenic potential, melanoma-initiating and melanoma-differentiation drivers, cell–cell adhesion molecules, and matrix metalloproteinases performed on MCAM and ABCB5 enriched CMCs, aimed at identifying those putative genes involved in early melanoma spreading and disease progression.

Overall, positive cells for MCAM, ABCB5, and both MCAM/ABCB5 could represent three different CMC subpopulations “endothelial,” “stem” phenotypes, or “hybrid stem–endothelial” phenotypes, respectively, which could display distinct gene expressions and exhibit distinct roles in early local invasion and metastases.

## Methods

### Patients and Healthy Donors

Patients’ demographic and clinical characteristics are shown in Table 1. A cohort of 22 melanoma patients entered prospectively this study. Information and consent forms, previously approved by the ethical local institutional review board (code: prot.0013157/2015), were provided at diagnosis, together with the permission to collect blood samples for research purposes. Patients were considered eligible if they had a histological and immunohistochemical (S-100, HMB-45, and MART-1) confirmed diagnosis of MM and if staged AJCC ≥ pT1b (Gershenwald and Scolyer, 2018). They were staged as follows: five patients (23.8%) in AJCC stage IB, three patients (14.3%) in AJCC stage II, five patients (23.8%) in AJCC stage III, and eight AJCC stage IV (38.0%). We stratified the 21 melanoma patients into two disease categories: early-AJCC-staged treatment-naive patients and clinically evident advanced-stage patients. The former included IB–II AJCC stages (8/21); the latter, III–IV AJCC stages (including three patients who showed cutaneous *in transit* metastasis without nearby lymph nodes involvement) (13/21). All patients were cured at the Dermatology Department of the University of Rome “Tor Vergata” (Italy). Twenty healthy donors from our Transfusion Center were included in the study as negative control population.

**TABLE 1:** Patients’ (pts) demographic and clinical characteristics.

**Sex**	**N°**	**%**

Female	10	/
Male	11	/
Age (years)	44 (mean)	24–84 (range)

**Primary tumor site**	**N°**	**%**
Head and neck	1	4.80
Trunk	11	52.4
Extremity	5	23.8
Unknown	4	19.0

AJCC* stage	**N°**	**%**
>IB	5	23.8
II: IIA (2); IIB (1)	3	14.4
III: IIIA (2); IIB (2) IIIC (1)	5	23.8
IV	8	38.0

**Time from diagnosis**	**(Years)**	
AJCC Early Stage I–II	0	/
AJCC Advanced Stage III**–IV	0–12	/

**Clinically disease free**	12	
**Clinically evident disease**	10	

*The AJCC (American Joint of Cancer Committee) staging was evaluated at the time of the blood draw after diagnosis of primary melanomas or diagnosis of first distant metastases in case of occult melanomas. **Three out of five pts showed cutaneous in transit metastasis without nearby lymph nodes involvement (N1c) (Gershenwald and Scolyer, 2018).

### Cell Lines

The selected gene-expression panel, with the exception of melanoma tissue stem and differentiation markers *TyrOH*, *MelanA/MART1*, and *ABCB5*, was previously tested and validated on 14 primary tumor cell lines including LnCap, DU145 (prostate cancer); MB 231, MCF7 (breast cancer); C33A, HeLa (cervix cancer); Mel 10, Mel 14, FO 1, Colo 38 (MM); SH-Sy5 (neuroblastoma); U87 (glioma); U266, Arp 1 (multiple myeloma), as partially reported in Table 2 (Rapanotti et al., 2018). The melanoma tissue stem and differentiation markers *TyrOH*, *MelanA/MART1* were tested on Mel 10, Mel 14, FO 1, Colo 38 (MM), as previously reported (Rapanotti et al., 2009, 2014). The fibroblast cell line EDS and the endothelial cell line HUVEC were included as positive and negative controls. Cell lines were grown in RPMI-1640 (GIBCO-BRL, Waltham, Massachusetts, MA, United States) supplemented with 10% fetal bovine serum (GIBCO-BRL) and antibiotics, in a humidified atmosphere with 5% CO_2_ at 37°C temperature. Cells were detached by trypsinization, then centrifuged, washed twice with phosphate-buffered saline, and stored at −70°C, until use.

**TABLE 2:** Analysis of expression of angiogenic factors, pro-angiogenic factors, cell-cell adhesion molecules, and Matrix-Metallo Proteinases in cell lines.

Cell line	Cell origin	VEGF	Ang2	MCAM/MUC-18	bFGF	VE-CADH	E-Cadh	N-Cadh	MMP-2	MMP-9
**EDS**	Fibroblast culture	NEG	NEG	NEG	NEG	NEG	POS	NEG	NEG	NEG

**HUVEC**	Endothelial culture	POS	POS	POS	POS	POS	POS	NEG	POS	POS

**Mel 10**	Melanoma	POS	POS	POS	POS	POS	POS	POS	POS	POS

**Mel 14**	Melanoma	POS	POS	POS	POS	POS	POS	POS	POS	POS

**FO 1**	Melanoma	POS	POS	POS	POS	POS	POS	POS	POS	POS

**Colo 38**	Melanoma	POS	POS	POS	POS	POS	POS	POS	POS	POS

**SH-Sy5y**	Neuroblastoma	POS	NEG	POS	POS	POS	NEG	POS	POS	POS

**LnCap**	Prostate cancer	POS	NEG	NEG	NEG	POS	POS	NEG	POS	POS

**DU 145**	Prostate cancer	POS	POS	POS	POS	POS	NEG	NEG	POS	POS

**U 87**	Glioma	POS	POS	POS	POS	POS	POS	POS	POS	POS

**MB 231**	Breast cancer	POS	POS	NEG	POS	POS	POS	NEG	POS	POS

**MCF-7**	Breast Cancer	POS	POS	NEG	NEG	POS	POS	NEG	POS	POS

**C33A**	Cervix Cancer	POS	POS	NEG	POS	POS	POS	NEG	POS	POS

**HeLa**	Cervix Cancer	POS	POS	POS	POS	NEG	POS	NEG	POS	POS

**U 266**	Multiple myeloma	POS	NEG	NEG	POS	POS	NEG	POS	POS	POS

**Arp 1**	Multiple myeloma	POS	NEG	POS	POS	POS	NEG	POS	POS	POS

### CMC Enrichment

Circulating melanoma cell enrichment was conducted on 21 melanoma patients. Fifteen milliliters of blood was collected 1 week after sentinel lymphadenectomy for the eight patients AJCC “early-staged” and at first clinical evidence of progression disease for the 13 patients AJCC “advanced-staged,” considered in both cases as baseline-blood-drawn. A home-made immunomagnetical fluorescein isothiocyanate (FITC)–conjugated anti-CD146, anti-ABCB5, and anti-CD146/antiCDABC5 antibodies selection preceded by CD45 immunodepletion allowed us to enrich three subpopulations based on expression of “endothelial” E-CMCs (CD45^–^MCAM^+^), “stem” S-CMCs (CD45^–^ABCB5^+^), and “hybrid stem-endothelial” E/S-CMCs (CD45^–^MCAM^+^/ABCB5^+^) markers. Before performing CD45 depletion, platelets and erythrocytes were removed by using density gradient (HetaSep/STEMCELL Technologies, Vancouver, Canada); enriched leukocytes were then conjugated with whole blood, with several stoichiometric and volume adjustments. The CD45 depletion was performed using the Blood CD45 Depletion Cocktail containing monoclonal CD45 antibody and magnetic nanoparticles (EasySep Magnetic Nanoparticles; STEMCELL Technologies) achieved using manufactory procedure. After CD45 depletion, each cell suspension was split in three separate vials and then underwent three CMC enrichments, using manual immune-magnetic beads FITC-conjugated to mouse anti-human MCAM/CD146 (BD Pharmingen^TM^, clone P1H12, Franklin Lakes, New Jersey, NJ, United States), to rabbit anti-human ABCB5 (Biorbyt^TM^, clone RB16781, Cambridge, United Kingdom), and contemporary to anti-human MCAM/CD146–anti-human ABCB5 (BD Pharmingen^TM^/Biorbyt^TM^) monoclonal antibodies. A total of 63 enriched samples were recovered. The enrichment protocol was designed in-house. The stoichiometry and experimental conditions for antibody-cell conjugation were established after having carried out serial dilutions of the melanoma cell lines into blood drawn sample from 1,000 to 1 cell/mL considering the expected rarity of CMCs in the blood stream (1–3 CMC/∼5 billion blood cells). We point out that for efficient antibody coupling, at least 10 mg of antibody was used per milligram of immune-magnetic beads. Patients were defined as CMCs-positive when MCAM^+^ or ABCB5^+^ but CD45^–^ nucleated cells were detected. We included blood samples from 20 healthy donors from our Transfusion Center as negative control population.

### Selection of Reference Gene Panel

We performed a qualitative test, that is, OneStep reverse transcriptase–polymerase chain reaction (RT-PCR) or RT-nested PCR in order to have a robust reference gene panel suitable for gene expression analysis of enriched CMC subpopulations. The candidate reference genes were selected from articles available in the PubMed databases and known to be strongly associated with angiogenic potential, melanoma-initiating and melanoma-differentiation, cell–cell adhesion molecules pathways, and matrix metalloproteinase extravasation: all processes are major key players in the regulation of EMT, early melanoma spreading, and disease progression. The 13 selected genes were as follows: vascular endothelial growth factor (*VEGF*); basic fibroblast growth factors (*bFGF*); vascular endothelial cadherin (*VE-Cadh CDH5*); endothelial antigen *MCAM/MUC18/CD146* isoforms long, short, and 5-portion; epithelial cadherin (*E-Cadh CDH1*); neuronal cadherin (*N-Cadh CDH2*); matrix metalloproteinases (*MMP-2*, *MMP-9*); tyrosinase (*TyrOH*); and melanoma antigen recognized by T cells *MelanA/MART1* and *ABCB5* (Lehmann et al., 1987; Ray and Stetler-Stevenson, 1994; Curry et al., 1996; Xie et al., 1997; Schittek et al., 1999; Silye et al., 1998; Carmeliet and Jain, 2000; Hazan et al., 2004; Frank et al., 2005; Melnikova and Bar-Eli, 2006).

### RNA Isolation and RT-PCR Methods

Total RNA was isolated from primary tumor cell lines and CMC subpopulations, using a home-made protocol based on Chomczynski and Sacchi (1987) protocol modified for poorly cellular samples. RNA integrity was measured for RNAs extracted from the 63 enriched melanoma patients subpopulations, the 16 cell lines, and the 20 healthy donors using the NanoDrop 2000 (ThermoFisher, Waltham, Massachusetts, United States) according to the manufacturer‘s instructions. RNA integrity was also checked electrophoretically.

Total RNA (Applied BioSystems, Roche Molecular Systems, Inc., Branchburh, NJ, United States) was used in all RT-PCR experiments, as indicated in the manufacturer’s instructions. First-strand cDNA was generated with 2.5 mM oligo d(T)_16_, 5 mM MgCl_2_, 1 mM dNTPs, 1 unit of RNase inhibitor (Applied BioSystems), and 1-h incubation at 42°C. Two microliter aliquots of cDNA were used for single-step sensitive RT-PCR for all genes, with the exception of *MCAM/MUC18/CD146*, *ABCB5*, *TyrOH*, and *MelanA/MART1* where nested PCR was also performed. A hot start Taq polymerase was used for amplification using the housekeeping gene β2-microglobulin as control. Cell line total RNAs have always been included as positive or negative controls in all performed experiments. Primer sequences and PCR conditions are reported in detail in the Supplementary Material. The resulting nested products (25 μL) were analyzed on a 1.8% agarose gel. All PCR experiments were always performed in triplicate. Contamination was evaluated by including no template control in all experiments.

### Statistical Analysis

For statistical evaluations, because of the small sample size, we stratified the 21 melanoma samples into two disease categories: early and advanced stages. The former includes IB–II AJCC stages (8/21); the latter, III–IV AJCC stages (13/21). Univariate analysis of relationship among correlations between biomarkers in subpopulations was performed using Spearman rank correlation test. SPSS 20.0 software program (SPSS Inc., Chicago, IL, United States) was used for statistical analysis.

## Results

### Expression of Melanoma-Initiating and Melanoma-Differentiation Drivers, Proangiogenic, Markers Cell–Cell Adhesion Factors, and Matrix Metalloproteinases in Cell Lines

In our previous studies, M14 melanoma cells were serially diluted to mimic *in vivo* conditions of occult metastatic melanoma cells in blood and establish the sensitivity of our assay, starting from 1 × 10^6^ M14 cells mixed with 7 × 10^6^ cells from blood of healthy donors (BHD) up to 1 M14 melanoma cell as already described (Rapanotti et al., 2009, 2014). In order to evaluate the level of detection, we performed serial dilutions of M14 melanoma cells in 6 mL blood from healthy donors, starting from 1 × 10^6^ M14 cells into 7 × 10^6^ cells from BHD, up to 1 M14 melanoma cell. Performing one-step or nested PCR, we brought the sensitivity down even before 1 or 10 single melanoma cells (Rapanotti et al., 2009, 2014). The expression of proangiogenic, cell–cell adhesion factors and matrix metalloproteinases (*VEGF*, *bFGF*,*VE-Cadh*, *E-CADH N-Cadh*, *MCAM/MUC18/CD146*, *MMP-2*, and *MMP-9*) was heterogeneous (Rapanotti et al., 2018; Table 2). Nevertheless, all four melanoma cell lines (M10, M14, FO1, and Colo38) expressed all aforementioned genes and the melanoma tissue stem and differentiation markers *TyrOH*, *MelanA/MART1*, and *ABCB5*. These molecular assays allowed us to ensure the efficiency, specificity, and sensitivity of our amplification procedures, considering the expected poor cellular samples.

### Expression of Melanoma-Initiating and Melanoma-Differentiation Drivers, Proangiogenic, Markers Cell–Cell Adhesion Factors, and Matrix Metalloproteinases in Enriched CMC Subpopulations

The 63 enriched CMC samples collected at first were divided into three distinct subpopulations (each group consisting of 21 samples) and, based on the MCAM and ABCB5 enrichment, were classified as “endothelial” E-CMCs (CD45^–^MCAM^+^), “stem” S-CMCs (CD45^–^ABCB5^+^), and a third hybrid biphenotypic population endothelial/stem E/S-CMCs (CD45^–^MCAM^+^/ABCB5^+^). All these cellular subsets were molecularly characterized by assessing the expression panel of the 13 aforementioned genes. All 63 enriched CMC samples expressed β2-microglobulin housekeeping gene. This is a tangible demonstration of high-quality cell recovery and total RNA extraction, despite the expected rare representations of cells.

Overall, 56 samples (84.8%) were found positive for expression of 1 of the 13 transcripts at least in one fraction from their blood (E-CMCs, S-CMCs, or E/S-CMCs). Twenty of twenty-one (95.2%) patients expressed at least one transcript in one of the three fractions. Fifteen patients showed detectable transcripts both in two of the three (71.4%) and in all three subpopulations (71.4%), suggesting that CMCs can be efficiently enriched and isolated by either MCAM/CD146 or ABCB5 cell surface markers.

In this respect, peculiar case of a patient staged AJCC IV, who died shortly after the blood sample, was found negative for all transcripts, but for the housekeeping gene control.

Molecular detection of our 13 transcripts, documented in all cases, divided into the three subpopulations, “endothelial” E-CMCs, “stem” S-CMCs, and “hybrid-endothelial/stem,” expressed as percentage was as follows:

*MCAM/MUC18/CD146* 5′-portion (38.0, 33.3, 33.8%), long isoform (61.9, 9.5, 61.9%), short isoform (47.0, 33.3, 57.1%); ABCB5 (9.5, 19.0, 14.2%); *VEGF* (4.8, 19.0, 14.2%); *bFGF* (23.8, 19.0, 23.8%), *VE-Cadh* (38.0, 38, 57.1%), *N-Cadh* (23.8, 19.0, 23.8%), *MMP2* (47.6, 33.3, 61.9%), and *MMP9* (38.0, 50.9.0, 66.0%), respectively. Tyrosine hydroxylase, the first melanoma-associated marker (*Tyr-OH*) to have been proposed for CMC detection (Curry et al., 1996), was absent in “endothelial” (CD45^–^MCAM^+^) and “hybrid-endothelial/stem” (CD45^–^MCAM^+^/ABCB5^+^) populations, while positive (14.2%) only in the “stem” fraction (CD45^–^ABCB5^+^), in both early and advanced AJCC stage groups.

Melanoma-associated antigen *MelanA/MART1* (Schittek et al., 1999), another highly regarded marker, was never detected in all 63 CMC samples; the same occurred for the main epithelial cell adhesion molecule E-cadherin, *E-Cadh*.

**FIGURE 1:**
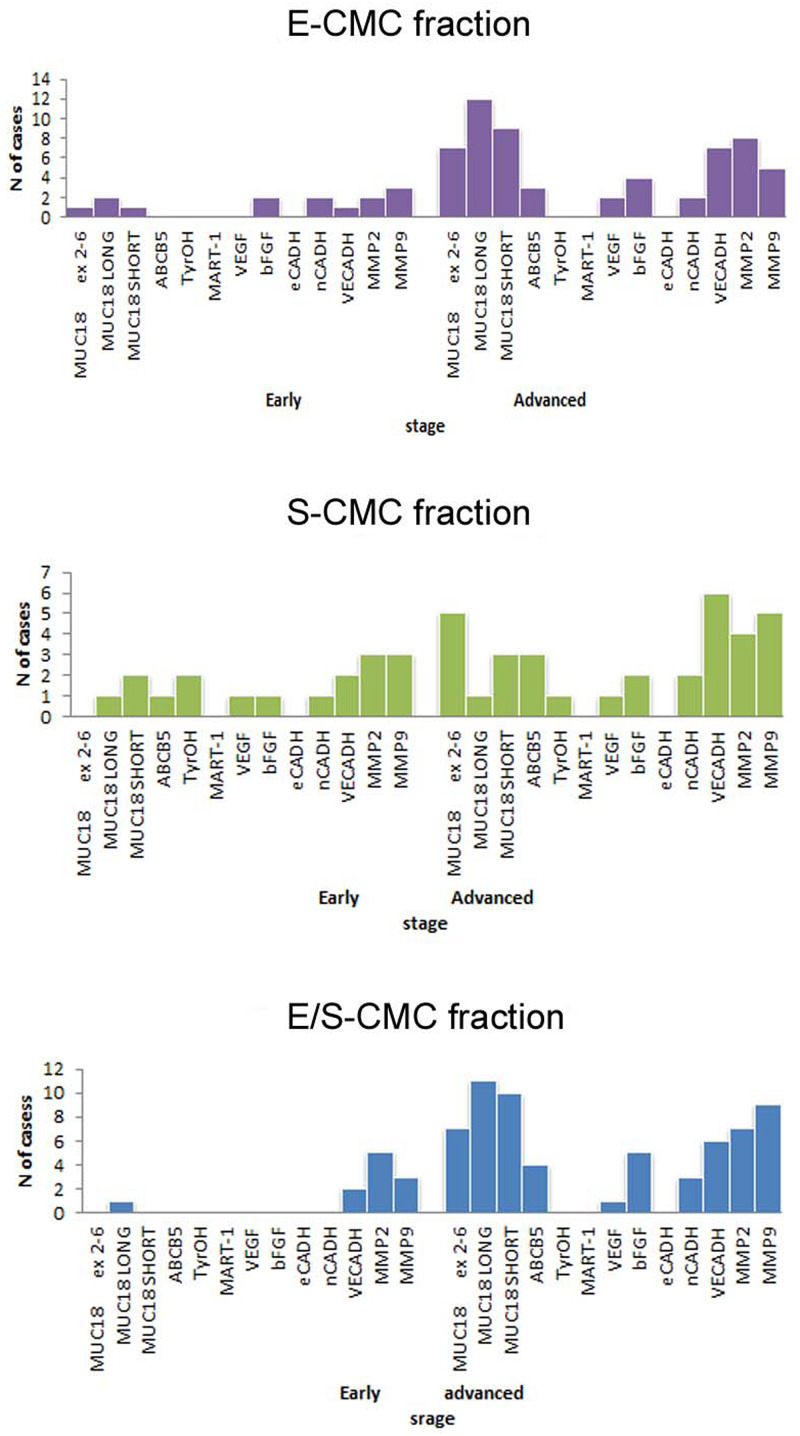
Bar graphs showed the distribution of analyzed genes in subpopulations selected for this study. Distribution of cases considering positivity status of all biomarker analyzed in the three different subpopulations (early: I–II AJCC-staged eight patients; advanced: III–IV AJCC -staged 13 patients).

The molecular analysis of the three subpopulations E-CMC fraction, S-CMC, and E/S-CMC in eight “early-AJCC-staged treatment-naive” patients and in 13 “advanced AJCC-staged clinically evident disease-patients” is reported in Table 2. As illustrated in Figure 1, the different expression of the selected genes was observed between and within CMC samples positive-enriched with MCAM-, ABCB5-, or MCAM/ABCB5-coated beads, suggesting the molecular heterogeneity of these three subpopulations in both AJCC-staged patient groups.

Overall, the advanced AJCC-staged clinically evident disease patients were characterized by higher gene expression compared to early-AJCC-staged treatment-naive, as expected (Table 3).

**TABLE 3:** Expression (percentage) of melanoma-initiating and melanoma-differentiation drivers, proangiogenic, markers cell-cell adhesion factors, and matrix-metallo-proteinases in three enriched CMC subpopulations from melanoma patients.

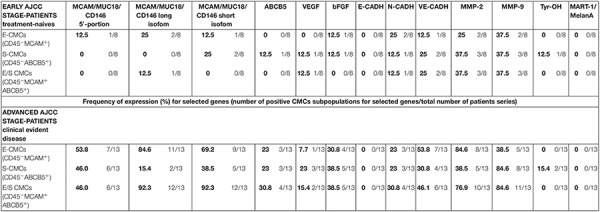

In early-AJCC-staged group, by comparing the two endothelial and stem subpopulations, we found that E-CMCs were characterized by the presence of *MCAM* 5′-portion and long isoform expressions in 12.5–25% and related to S-CMCs where it was absent (0%). On the other hand, the expression of *ABCB5*, *Tyr-OH*, and *VEGF* was found exclusively in S-CMCs fraction with respect to E-CMC fraction (0%). Both subpopulations showed heterogeneous expression for *short MCAM* isoform, *bFGF*, *N-CADH*, and *MMPs* (frequency range = 12.5–37.5%). The third “hybrid-endothelial/stem” fraction resulted into another distinct fraction, characterized by the absence of *MCAM* 5′-portion, *short MCAM* isoform, ABCB5, bFGF expression (0%), and a heterogeneous frequency for the long *MCAM* isoform, *VEGF*, *N-Cadh*, *Ve-Cadh*, *MMP2*, and *MMP9* (frequency range = 12.5–37.5%).

In the advanced AJCC-staged group, considered as a “positive control,” we documented an increased overall gene expression: particularly *MCAM* (*MCAM* 5′-portion, long and short isoforms) and *VE-Cadh* expressions that significantly increased in the E-CMC and hybrid enriched E/S -CMC (range = 46.1–84.6%), whereas VEGF and bFGF showed a low and moderate expression increase, respectively (frequency range = 15–38.5%). All these data also showed a number of positive CMC subpopulations for selected genes (Figure 1 and Table 3). No expression of these 13 genes was detected in E- (endothelial), S- (stem), and hybrid E/S-enriched fractions derived from similarly evaluated healthy donors (*n* = 20).

### Relationship Between Melanoma-Initiating and Melanoma-Differentiation Drivers, Proangiogenic, Markers Cell–Cell Adhesion Factors, and Matrix Metalloproteinases and Enriched CMC Subpopulations

Despite our smaller case series, in the early-AJCC stage group (eight patients), we could only emphasize a direct correlation between MMP2 and MMP9 biomarkers in E-CMC and hybrid E/S-CMC subpopulations (*p* < 0.05).

Here, we reported the most significant relationships for the purpose of this preliminary study in advanced-AJCC stage group (13 patients). For E-CMC subpopulation, we highlighted the following different statistically significant positive correlations among biomarkers in advanced AJCC stage group: *MCAM/MUC18/CD146* long and short isoforms (ρ = 0.59 and 0.79, *p* < 0.05); *MCAM/MUC18/CD146* 5′-portion, long and short isoforms, and *VE-Cadh* (ρ = 0.40, 0.48, and 0.66; *p* < 0.05); *ABCB5* (ρ = 0.50; *p* < 0.05); *VEGF* and *VE-CADH* (ρ = 0.68 and ρ = 0.48, respectively). In S-CMC subpopulation, we emphasized correlations between *MCAM/MUC18/CD146* long isoform and *N-Cadh* (ρ = 0.68) and between *VE-Cadh* and *TyrOH* (ρ = 0.56) and *MMP2* (ρ = 0.56). Direct correlations between *MMP9*, *MMP2* (ρ = 0.76), and *bFGF* (ρ = 0.569) were also reported. In the third hybrid E/S–CMC subpopulation, we demonstrated a consistent relationship between *MCAM/MUC18/CD146*, 5′-portion, long and short isoforms, and AJCC stages (ρ = 0.71, 0.76, and 0.89). *MCAM/MUC18/CD146* 5′-portion presence correlated directly with *VE-Cadh* (ρ = 0.53) and *MMP9* (ρ = 0.71) positives. All correlations were statistically significant at *p* < 0.05. We used Spearman correlation test.

## Discussion

Various cell surface antigens have been involved in the pathway of human melanoma metastases (Murakami et al., 2004; Medic et al., 2007; Rodic et al., 2014). Metastatic process involves altered and dysregulated processes of adhesion, migration, invasion, and proliferation of cancer cells that implicate cytokine receptors, adhesion molecules, and drug resistance–related antigens. Proteins associated with stem and progenitor cells are also detected in melanoma and include cancer testis antigens (Simpson et al., 2005; Velazquez et al., 2007), bone morphogenetic proteins (Hsu et al., 2008; Rothhammer et al., 2007), Notch receptors (Balint et al., 2005), Wnt proteins (Weeraratna et al., 2002), or specific stem cell–associated markers, such as multidrug resistance transporters of the ABC family, CD133, CD166, CD34, Nestin, and c-Kit 2 (Frank et al., 2005; Laga et al., 2010). Most of these studies employed melanoma cell lines, and only a few analyzed expression of cell surface antigens on human melanomas (Radford et al., 1996; Selzer et al., 2000; Djerf et al., 2009). Herein, we described how CMC subpopulations expressing MCAM, as melanoma-associated antigen (Xie et al., 1997) and/or ABCB5, as melanoma-initiating marker (Frank et al., 2005), display distinct gene profiles. We selected MCAM/MUC18/C146 not only because it is an effective marker to capture and detect CMCs, because of its high surface expression, up to 80% (Lehmann et al., 1987; Xie et al., 1997; Cristofanilli et al., 2004; Melnikova and Bar-Eli, 2006; Cohen et al., 2008; de Bono et al., 2008; Rapanotti et al., 2009, 2013, 2014, 2017; Quintana et al., 2010), but also because it is considered an EMT inducer (Shih, 1999; Bardin et al., 2001). In addition to its function as key oncogene in driving melanoma progression and metastasis, this membrane glycoprotein is a component of the interendothelial junction (Delorme et al., 2008) and is even recognized as a mesenchymal marker (Guezguez et al., 2007). Circulating melanoma cells have an important role in the interaction with bone marrow stromal cells that allow motility and migration of hematopoietic microenvironment to heterotopic sites (Sacchetti et al., 2007). Recently, an MCAM/MUC18/C146 stromal/mesenchymal signature has been described associated with poor prognosis in several cancers, in particular in breast cancer. Circulating tumor cell association to triple-negative receptor status promotes undifferentiated malignant cell motility (Zabouo et al., 2009; Onstenk et al., 2015; Stalin et al., 2017). In cancer, the EMT is associated with tumor stemness, metastasis, and resistance to therapy. The importance of cell plasticity in driving the transition of poorly tumorigenic epithelial carcinoma cells into highly aggressive stem CTCs via induction of an EMT has been well described. Relatively little is known about the specific cell states along the “E-to-M spectrum” in which stem CTC populations reside and the role that cell plasticity plays in enabling them to function effectively as tumor-initiating cells. It has recently defined that EMT occurs through distinct intermediate states with different invasive, metastatic, and differentiation characteristics (Bracken et al., 2008; Burk et al., 2008; Kalluri and Weinberg, 2009; Thiery et al., 2009; Yu et al., 2013; Jolly et al., 2015; Pastushenko et al., 2018; Kröger et al., 2019; Zhao et al., 2019). This network, from the molecular point of view, is composed of two gene pathways, SNAIL and ZEB, and two miR families, miR-200 and miR-34 (Bracken et al., 2008; Burk et al., 2008). The epithelial phenotype corresponds to high levels of miR-200 and miR-34, whereas the mesenchymal phenotype corresponds to high levels of ZEB and SNAIL. An experimental model provided direct evidence that residence in a hybrid E/M state was sufficient for maintenance of stem cell properties (Epithelial/Mesenchimal). Analysis of a large panel of cell surface markers (EpCAM, CD44, CD51, CD61, CD104, CD105, CD106), by combinatorial multicolor FACS, revealed the existence of multiple tumor subpopulations associated with different EMT stages in skin and solid cancers: from epithelial to completely mesenchymal states, passing through intermediate hybrid states. These cells share a mixed epithelial (e.g., adhesion) and mesenchymal (e.g., migration) phenotype, which thereby allow them to move collectively, as proposed by Jolly et al. (2015), reaching efficiently the bloodstream intact, giving rise to clusters of CTCs so forming metastases. Although all EMT subpopulations present similar tumor-propagating cell capacity, they display differences in cellular plasticity, invasiveness, and metastatic potential. In addition, it has been shown that these different EMT states are localized in different microenvironments and in contact with different stromal cells. Isolation of these CTC clusters and testing them along the E–M spectrum have become the most promising diagnostic approach in the clinic. However, it must be underlined that these reports have mainly analyzed CTCs derived from epithelial cancers characterized by cytokeratins and/or EPCAM (Epithelial Cell Adhesion Molecule), antigens commonly expressed and used for their isolation and detection. By contrast, CMCs do not commonly express these markers, because melanocytes originate from the neural crest lineage, and to date, it is not well formalized an ubiquitary melanoma cell surface marker. Nonetheless, what is known in melanoma, is that EMT is similarly characterized by loss of typical melanocytic histologic features, including apical-basolateral polarization, basement membrane integrity, and cell–cell adhesion, and acquisition of a more invasive phenotype (Pearlman et al., 2017).

Several findings documented that tumorigenic heterogeneity within the melanoma VGP is the definition of a subpopulation of human melanoma cells that express the multidrug resistance transporter known as adenosine triphosphate–binding cassette subfamily B, ABCB5 (Frank et al., 2005; Klein et al., 2007; Schatton et al., 2008; Laga et al., 2010). The ABCB5 transmembrane transporter, belonging to the superfamily of integral membrane proteins, is associated with melanomagenesis, stem cell maintenance, metastasis, and chemoresistance (Schatton et al., 2008). Human tumorigenic melanoma reveals that a minority of cells expresses the ABCB5 cell membrane–associated transporter. These cells tend to display a primitive molecular profile and correlate with clinical melanoma progression as determined by high-density tissue microarrays that allowed the screening of numerous melanomas representing progressive evolution from RGP to VGP and metastatic disease (Elliott and Al-Hajj, 2009; Laga et al., 2010). The association of an ABCB5-expressing melanoma subset with tumorigenic growth, typical of the VGP, supports the rationale that CMCs derive from rare cancer subpopulations that may potentially initiate metastases (Schatton et al., 2008; Civenni et al., 2011; Wilson et al., 2014).

Because the initiation and progression of vertical growth in human melanoma, often accompanied by phenotypic changes enabling greater cell motility and migration, are fundamental for melanoma progression, we decided to enrich and analyze CMCs from melanoma patients staged AJCC ≥ pT1b. At first, we developed a highly effective home-made CMCs enrichment protocol, selecting MCAM/MUC18/CD146 and ABCB5 as melanoma-specific epitopes, followed by molecular qualitative reference gene panel suitable to identify those genes that could provide great potential and biological information to better define melanoma high-risk and low-risk patients. The most significant finding from our study is that, based on gene expression data, MCAM/MUC18/CD146 and ABCB5 are suitable and effective cell-surface targets in liquid biopsy procedures. A differential expression of the specific transcripts was documented between and within the CMC fractions enriched with MCAM-, ABCB5-, and both MCAM/ABCB5-coated beads, confirming the consistency of our approach. The absence of molecular gene expression in our panel observed in healthy donors’ blood samples further validated our enrichment approach. This study confirms the phenotypic and molecular heterogeneity observed in melanoma CMCs (Klinac et al., 2014; Luo et al., 2014; Hong et al., 2018) and highlights genes that may be associated with the biology of these subpopulations and with melanoma progression. Another significant finding from this study is that, based on gene expression data, E-CMC, S-CMC, and E/S-CMC are three distinct subpopulations. This consideration is based on the only partial overlap detected among the gene expressions found in the three enriched CMCs fractions. In the early-AJCC-staged melanoma group, we exclusively detected in the enriched endothelial fraction E-CMC, *MCAM* expression, 5′-portion, and both isoforms, associated with *VE-CADH*, *MMP2*, and *MMP9* (these last genes mostly expressed). These findings indicated that MCAM, as melanoma-associated marker involved in heterotypic cell adhesion and tissue invasion of melanoma cells, characterizes the E-CMCs equipped with tumorigenic capabilities, such as migration and invasion.

Opposite to the MCAM-enriched CMC fractions, the ABCB5-enriched CMCs confirmed the rare *ABCB5* expression (Velazquez et al., 2007), found only in S-CMC fraction, when analyzing early-AJCC-staged patients. ABCB5, as melanoma-stem marker of slow-cycling population of tumor cells with self-renewal differentiation and proliferation capabilities, characterizes the S-CMC subpopulation, the only fraction that showed *ABCB5*, *TyrOH*, and *VEGF* expressions. The hybrid E/S-CMC fraction, despite the contextual MCAM and ABCB5 enrichment, does not express *ABCB5*, only showing the *MCAM/MUC18/CD146* long isoform associated with *VE-Cadh* and *MMPS* expressions. These findings suggest that these cells are “hybrid equipped with tumorigenic capabilities,” such as motility, migration, and invasiveness, as they travel through the bloodstream.

We believe that the first purpose of our work can be considered achieved, because we could define, by assessing a robust qualitative gene-expression panel, those genes suitable to identify early-AJCC-staged patients but carrying more aggressive CMCs. These data have been confirmed when analyzing blood samples from advanced AJCC-staged patients, interpreted as positive control, a statistically significant increase of *MCAM/MUC18/CD146*, *ABCB5*, *MMPs*, and *VE-Cadh* expressions was shown. In parallel, all three distinct CMC fractions in both AJCC-staged patients groups did not show a very high molecular positivity of proangiogenic factors, *VEGF* and *bFGF.* Even the *N-Cadh* expression was not highly expressed, despite its well-known association with neuroectodermal malignant tissue transformation, suggesting that we selected three subpopulations sharing undifferentiated phenotypes. This consideration is even strengthened by the absence of *MART1/MelanA* and *E-Cadh*. Particularly, the loss of E-Cadh–mediated adhesion characterized the transition from benign lesions to invasive and metastatic cancer, associated with EMT. Downregulation of molecular expression of *E-Cadh*, if considered as a tumor suppressor gene, allows and enhances the invasion of adjacent normal tissues, increasing the metastatic potential.

We believe that this qualitative molecular expression analysis performed on these enriched MCAM/MUC18/CD146 and/or ABC5 CMCs provides evidence that these are three distinct CMC subpopulations, sharing primitive, “stem-mesenchymal” behavior, which makes them highly aggressive and able to metastasize. Despite our smaller case series, this study is among the first (Gutiérrez garcía-rodrigo et al., 2017; Aya-Bonilla et al., 2019; Zhao et al., 2019), to characterize different cancer subpopulations, contemplating a hybrid fraction, unveiling the molecular expression and suggesting distinct biological pathways activated in these cells. Molecular expression analysis especially of *MCAM/MUC18/CD146*, *MMPs*, and *VE-Cadh* could provide great potential and biological information to better define more aggressive CMC subpopulations and provide useful evidence to determine a suitable clinical approach, when analyzing early-AJCC-staged patients.

Our observations need to be validated on a larger case series. Particularly, we need to extend the early-AJCC-stage cohort of patients to achieve a stronger statistical significance. Finally, quantitative real-time PCR should be assessed for at least *MCAM/MUC18/CD146*, *MMPs*, and *VE-Cadh* to further validate the prognostic role of these reference genes.

## Data Availability Statement

All datasets generated for this study are included in the article/Supplementary Material.

## Ethics Statement

The studies involving human participants were reviewed and approved by the Ethical Commitee University of Rome Policlinico “Tor Vergata” number prot.0013157/2015. The patients/participants provided their written informed consent to participate in this study.

## Author Contributions

MR conceived and designed the study and organized the database. MR and LB wrote the first draft of the manuscript. MR, EC, PR, SB, and LB substantially contributed to the conception, designed the work, and analyzed data. TS performed all molecular biological experiments. EC, GS, PR, and AO as dermatology clinicians, anatomo-pathogist, and surgery, respectively, selected, diagnosed, cured and collected all the clinical informations. GC performed the statistical analysis and wrote the related section of manuscript. MR, EC, AO, PR, PV, SB, and LB revised it critically for important intellectual content. All authors contributed to manuscript revision, read and approved the submitted version.

## Conflict of Interest

The authors declare that the research was conducted in the absence of any commercial or financial relationships that could be construed as a potential conflict of interest.
