# Supplementary material for: Stem–Mesenchymal Signature Cell Genes Detected in Heterogeneous Circulating Melanoma Cells Correlate With Disease Stage in Melanoma Patients
Source: Front Mol Biosci. 2020 May 29;7:92. doi: 10.3389/fmolb.2020.00092 (PMC7272706; doi:10.3389/fmolb.2020.00092)
Supplement: Supplementary file 1 [file Data_Sheet_1.pdf]

PRIMERS	SEQUENCES	Tm ANNEALING	CYCLES
§Beta-2 Micro sense<br>§Beta-2 Microantisense	5'-ACC CCC ACT GAA AAA GAT GA-3 '<br>5'-ATC TTC AAA CCT CCA TGA TG-3'	58°C- 1 min	40
§ VEGF sense<br>§ VEGF antisense	5'-TCGGGC CTC CGA AAC CAT GAA CT-3'<br>5'-TCC TGG TGA GAG ATC TGG TTC CC-3'	64°C- 1 min	40
§ bFGF sense<br>§ bFGF antisense	5'-AGC AGA AGA GAG AGG AGT TGT GTC -3'<br>5'-CCC AGG TCC TGT TTT GGA TCC AAG-3'	60°C- 1 min	40
*MMP-2 sense<br>*MMP-2 antisense	5'-CCTGCCCCTCCCTTCAACCA-3'<br>5'-GTTTCCGCTTCTGGCTGGGTC-3'	64°C- 1 min	 40
*MMP-9 sense<br>*MMP-9 antisense	5'-CCTGCCCCTCCCTTCAACCA-3'<br>5'-GTTTCCGCTTCTGGCTGGGTC-3'	59°C- 1 min	40
*E-cadherin sense<br>*E-cadherin antisense sense	5'-GAC CAG GAC TAT GAC TAC TTG AAA CG -3'<br>5-ATC TGC AAG GTG CTG GGT GAA CCT-3	56°C- 1 min	40
* VE-cadherin sense<br>* VE-cadherin antisense sense	5'-CACTGGAACCCCCACAGGAAAAGA-3'<br>5'-GGACAGCGTTCTCACACACTTTGG-3'	58°C- 1 min	40
§ MCAM/MUC18 sense<br>§ MCAM/MUC18 antisense<br>§ MCAM/MUC18 nested sense<br>§ MCAM/MUC18 nested antisense<br>§ MUC 18 long sense<br>§ MUC 18 long sense<br>§ MUC 18 long NES sense<br>§MUC 18 long NES antisense<br>§ MUC 18 short sense<br>§ MUC 18 short antisense<br>§ MUC 18 short NES sense	5'-CCA AGG CAA CCT CAG CCA TGT-3<br>5'-CTC GAC TCC ACA GTC TGG GAC GAC-3'<br>5'-GTC ATC TTC CGT GTG CGC CA-3'<br>5'-GTA GCG ACC TCC TCA GGC TCC TTA-3'<br>5'-CCC TCA CAC CAG ACT CCA AC-3'<br>5'-GTT CGC TCT TAC GAG ACG GG-3'<br>5'-GGT CAT CGT GGC TGT GAT TG-3'<br>5'-GTA GCG TGA TCT CCT GCT TCC-3'<br>5'-TCA TAC CAG AGC CAA CAG CA-3'<br>5'-CTC TCC ATC TCC TGC TTC CC-3'<br>5'-AGA GAG AAA GCT GCC GGA G-3'	52°C- 1 min<br><br>52°C- 1 min<br><br>57°C- 1 min<br><br>60°C- 1 min<br><br>58°C- 1 min<br><br>60°C- 1 min	40<br><br>30<br><br>40<br><br>30<br><br>40<br><br>30
§ TYR-OH sense<br>§ TYR-OH antisense<br>§ TYR-OH nested sense<br>§ TYR-OH nested antisense<br>§ MART 1 sense<br>§ MART 1 antisense<br>§ MART1 NES sense<br>§ MART1 NES antisense	5'TTG GCA GAT TGT CTG TAG CC-3'<br>5'AGG CAT TGT GCA TGC TGC TT-3'<br>5'GTC TTT ATG CAA TGG AAC GC-3'<br>5'GCT ATC CCA GTA AGT GGA CT-3'<br>5'TGA CCC TAC AAG ATG CCA AG-3'<br>5'TCA GCA TGT CTC AGG TGT CT-3'<br>5'TCA TCT ATG GTT ACC CCA AG-3'<br>5'TCA TAA GCA GGT GGA GCA T-3'	55°C- 1 min<br><br>55°C- 1 min<br><br>55°C- 1 min<br><br>55°C- 1 min	40<br><br>30<br><br>40<br><br>30
*ABCB5 sense<br>*ABCB5 anti sense<br>*ABCB5 sense<br>*ABCB5 anti sense	5'GATGAACAGATGGAGTCAATG-3'<br>5'CTGCCGTAAAATAATCCCTGC-3'<br>5'GCTGAGGAATCCACCCAATCT -3'<br>5'CACAAAAGGCCATTCAGGCT-3'	55°C- 1 min<br>55°C- 1 min	40<br><br>30

Supplemental File. Primer sequences and PCR conditions. Sequences were reported § ( ) or designed * from the predicted sequence using the Gene Scan program (http://genes.mit.edu/genscan.HTML) § Hoon DS, Wang Y, Dale PS, Conrad AJ, Schmid P, Garrison D et al. Detection of occult melanoma cells in blood with a multiple-marker polymerase chain reaction assay. *J Clin Oncol* 1995; **13**: 2109-2116.
